# Intelligent Scheduling for Underground Mobile Mining Equipment

**DOI:** 10.1371/journal.pone.0131003

**Published:** 2015-06-22

**Authors:** Zhen Song, Håkan Schunnesson, Mikael Rinne, John Sturgul

**Affiliations:** 1 Department of Civil and Environmental Engineering, School of Engineering, Aalto University, Espoo, Finland; 2 Division of Mining and Geotechnical Engineering, Department of Civil, Environmental and Natural Resources Engineering, Luleå University of Technology, Luleå, Sweden; 3 School of Civil, Environmental and Mining Engineering, University of Adelaide, Adelaide, Australia; China University of Mining and Technology, CHINA

## Abstract

Many studies have been carried out and many commercial software applications have been developed to improve the performances of surface mining operations, especially for the loader-trucks cycle of surface mining. However, there have been quite few studies aiming to improve the mining process of underground mines. In underground mines, mobile mining equipment is mostly scheduled instinctively, without theoretical support for these decisions. Furthermore, in case of unexpected events, it is hard for miners to rapidly find solutions to reschedule and to adapt the changes. This investigation first introduces the motivation, the technical background, and then the objective of the study. A decision support instrument (i.e. schedule optimizer for mobile mining equipment) is proposed and described to address this issue. The method and related algorithms which are used in this instrument are presented and discussed. The proposed method was tested by using a real case of Kittilä mine located in Finland. The result suggests that the proposed method can considerably improve the working efficiency and reduce the working time of the underground mine.

## Introduction

In order to improve the profit and recover the investment of a mine, it is important to optimize the mining process. It is also important to keep the process in an optimal/near-optimal manner to obtain the target of the overall mine plan. Several studies have been carried out to improve the operational performance for underground mining, especially to optimize the loader-trucks cycle. Weintraub et al. [1] used a linear programming-based heuristic approach to multiple trucks with various capacities to minimize queuing time at loading points. According to Weintraub, this resulted in about 8 percent increase in productivity at the Chuquicamata mine in Chile. White and Olson [2] studied a truck-dispatching system based on a combination of network models, linear programming, and dynamic programming. Their study aimed to maximize production, minimize material handling, and guarantee blending constraints. The method is used to determine mass flows along paths. This system has been running in real-time applications in more than 10 mines and has increased their productivity by 10~20 percent. Beaulieu and Gamache [3] presented an enumeration algorithm based on dynamic programming to optimally manage the fleet in underground mines. This method arranges vehicle movement in a haulage network which is composed of one-lane bidirectional roads. It aims to find the route and schedule for each machine to minimize the moving time and to be free of conflicts. The authors explain the state transfer from an initial/previous state of the machine to a new state, and the propagation of states. Many examples are given using this approach for underground mining and other industrial environments. Saayman et al. [4] proposed an autonomous vehicle dispatch system for a diamond mine using block caving method. This hybrid system consists of discrete and continuous state. Collision avoidance is considered for underground mining, and four scenarios for avoidance are illustrated. The objective is to maximize the productivity and profit. Five different dispatching strategies for one week’s production are evaluated using a simulated environment in MatLab. Most of the results indicate that improvements can be possible based on current methods. McKenzie et al. [5] provided a method to optimize the location of a feeder or a conveyor. Break-even was the proximity of the feeder to the mine vs. the time spent to relocate the feeder, as mining moves forward. The problem has been solved as a shortest-path model by dynamic programming. According to McKenzie, implementing the method leads to a reduction of about 14 percent of operational cost at the mine. Nehring et al. [6] proposed a short term scheduling model for production scheduling and loader-truck allocation in conceptual Kelvin underground operations. The objective is to minimize deviation from targeted metal production. The model is designed to schedule loader-truck movements and Mixed Integer Linear Programming (MILP) is used to find out the optimal results. The shift-based schedule can be optimized and loader-trucks can be rapidly reassigned as underground operating conditions change. Optimal results are generated within minutes when a conceptual dataset was tested. Yu et al. [7] studied how to solve an vehicle routing problem by using fast local search and parallel computing of a genetic algorithm, in order to minimize the number of vehicles and the total transport distance or time. This method does not only improve the ability of optimization in a global scale, but also ensures the effectiveness of operation in the Zhengzhou coal mine in China.

**Fig 1:**
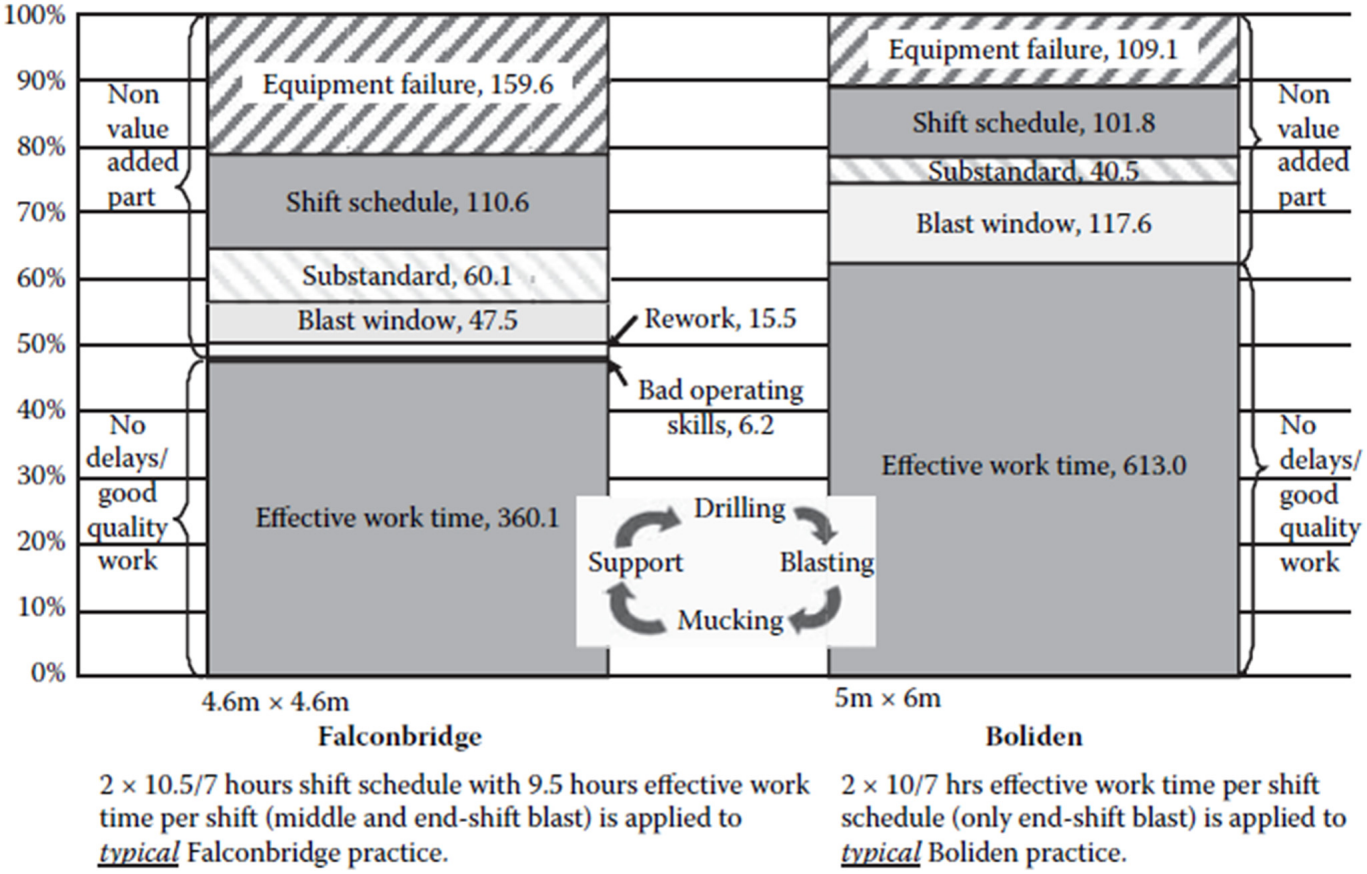
Time delay impacts for typical excavation practices with drilling and blasting [8].

However, there have not been relevant studies that cover the entire mining process of underground mining. Normally, underground mining operations are much more complicated and diverse compared with surface mining. The difference is mainly caused by mining methods, and the complexity is due to additional constraints required for underground mining. In underground mining operations, mobile mining machines are mostly scheduled instinctively, without theoretical support for these decisions. It can cause less confidence for miners and less efficient operations. Additionally, foremen in each shift may change the schedule based on their own experiences, if they do not agree with the existing schedule. It is difficult to keep the operations consistent. Furthermore, in case of unexpected events, it is hard for miners to rapidly find solutions for a new schedule and to adjust the operations. Therefore, there is a need to develop a decision support instrument which can help miners to initiate the schedule of mobile mining equipment, and rapidly reschedule these machines when unexpected events occur. Fig 1 shows that scheduling (incl. re-scheduling) can be a considerable part (more than 10%) of total working time, even in modern mines, such as Falconbridge and Boliden.

With the development of Information and Communications Technology (ICT), there are increased demands for improving the operation performance of underground mining process. Real-time monitoring and scheduling for underground mining should be managed optimally and promptly, as is commonly done for surface mining operations. It is likely that rapid gathering of operating information and intelligent application are the norm for future mines. Today, we can find many footprints of ICT in mining operations, both for surface or underground, e.g., communication and monitoring. However, there are not any intelligent applications for scheduling various operations in the underground mining process. This study is committed to develop a mine-wide decision support instrument to schedule the mobile mining equipment and improve the overall performance of the entire underground mining process.

## Proposed Method

**Fig 2:**
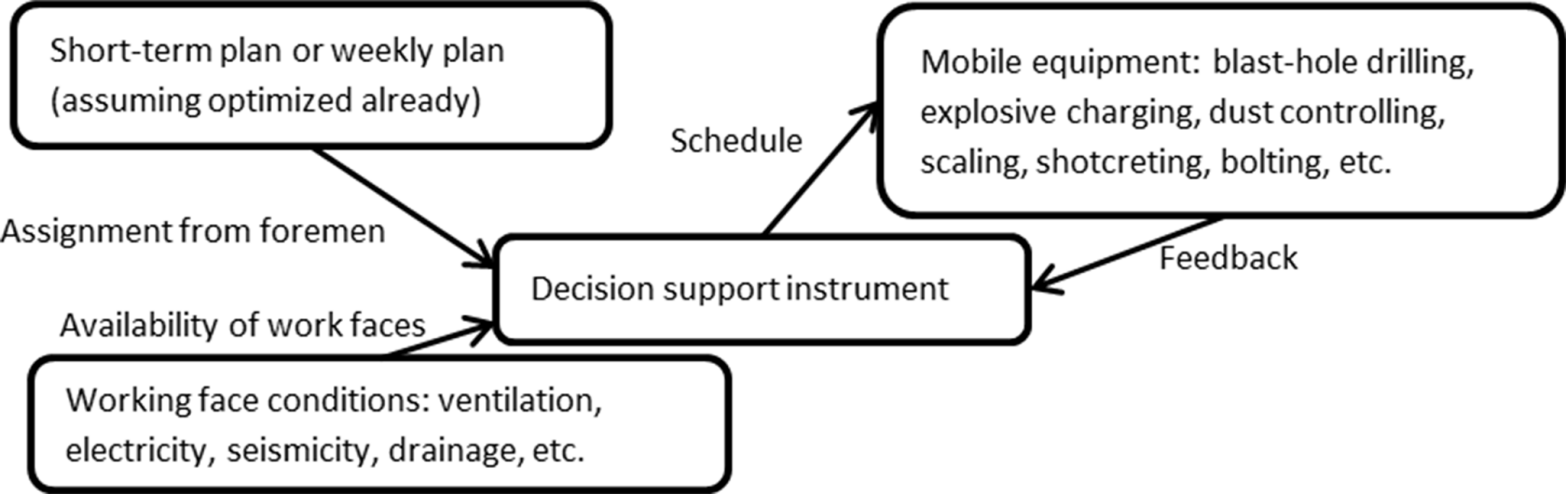
Relations of the instrument and its environment.

The decision support instrument should be able to initiate schedules of mobile mining equipment for mine managers on a shift-to-shift basis, as well as propose solutions of new schedule when encountering unexpected events that can disturb underground mining. These events can consist of, but are not limited to: seismicity, rock failure, underground flood, shortage of power/air/water at working faces, breakdown of equipment (including mobile and immobile equipment), etc. Most control systems of immobile equipment (e.g. crushing, conveying, hoisting, and ventilation) have been configured according to their manufacturers specifications. These commercially available control systems are normally interfaced with computers and customized control loops. Since they are usually being configured at an optimum working condition during the commissioning of the equipment, it is not recommended to make changes or optimize during every shift. Therefore, the overall performance of mining process would be improved by optimizing the scheduling of the mobile mining equipment (e.g. drill rigs, shotcreters) by adapting the constraints of the above control systems and other underground conditions. Fig 2 gives the basic perspective of how the schedule optimizer interacts with its environment. The schedule optimizer will first gather the work plans from foremen, the availability of working faces, and the status and performance of equipment. Then it will schedule the mobile mining equipment. The scheduling aims to use the mobile mining equipment as optimally as possible to achieve the short-term mining plan as productive and energy-efficient as possible.

**Fig 3:**
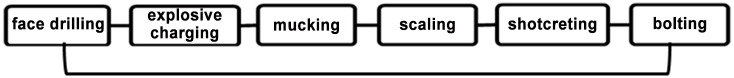
Operations of mobile mining equipment in mine excavation.

**Fig 4:**
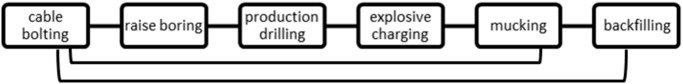
Operations of mobile mining equipment in mine production.

Underground mines consist of various operations. The complexity of the operations can considerably affect how easy it is to implement the technique and how useful the output is. It is crucial to define the proper scope we are aiming for. Firstly, the use of the technique of the schedule optimization is confined to the operations in hard rock mining. Coal mining as a continuous method already has technical solutions for automated operation. Secondly, the technique is supposed to be applicable for all underground hard rock mining methods. Thirdly, exploration and closure obviously do not relate much to day-to-day mine operations. In most cases, excavation and production take place simultaneously, and most of the mobile mining equipment is used for these two stages. Therefore, the scheduling should cover the operations of excavation and production, except the mucking operation because there are software applications available for it. Without loss of generality, the common processes are considered at working face respectively for excavation and production are shown in Figs 3 and 4.

### 2.1 Inputs to the Instrument

#### 2.1.1 Input from Mine Plans

In general, the long-term mining plan and the short-term mining plan interact with each other. One needs to be updated with the changes in the other. The exact duration of long-term and short-term are not clearly defined in practice, because their meanings are different for each mine. Under a short-term plan, normally there are weekly plans that provide mine operators with more details to achieve the goals set by the short-term plan. Furthermore, a weekly plan is distributed to the department of operation. The weekly plan mainly consists of two working contents: heading (i.e. working face where excavation takes place) and stope (i.e. working face where production takes place). The heading part tells foremen how many metres are planned to be excavated this week, while the stope part tells the foreman how many tons from which stope are planned to be mined this week. The input from mine plans should include the names of headings and stopes, their respective workloads (heading lengths and stope tonnages), and their spatial relations (distance between two working faces).

#### 2.1.2 Input from Working Faces

The input from the working face can be categorized in two groups. One group has Boolean values from electricity, water, air, drainage, ventilation and seismicity which can determine whether headings and stopes are available for excavation and production. The other group has boundary values, e.g. crusher feed rate, conveyer payload, and stockpile capacity which can mainly constrain the operating rate.

#### 2.1.3 Input from Mobile Mining Equipment

The development of ICT enables the establishment of interactions between the decision support instrument and mobile mining equipment. The mobile mining equipment should report the current status and receive orders proposed by the instrument as shown in Fig 2. The status of equipment can be available (idle) and unavailable (busy, repair, etc.), and the unavailable time of repair should be estimated by operators. To propose an optimal schedule to foremen, the instrument needs many working parameters from mobile machines, e.g. travelling speeds, drilling time per blast-hole, charging time per blast hole, scaling time per m^2^, shotcreting time per m^2^, curing time, and bolting time per bolt, etc. It is noted that there are normally travelling speeds and operating rates recommended by manufacturers for underground mining. It is ideal to obtain the working parameters by statistical analysis from historic data. In practice, it is also important to estimate working parameters by experienced operators.

### 2.2 Output from the Instrument

The output produced by the instrument should be useful to assist decision making and schedule mobile mining machines for short term. The results should be simple, compact, rapidly available, and easy to understand for miners. They should be integrated in a Gantt chart showing when a specific machine should work on a specific working face. The output can assist foremen in initiating a schedule and renewing the schedule when encountering disturbances in underground mining operations.

### Algorithms and Examples

Algorithms are the core part in the schedule optimizer. The inputs are actually constraints. It is noted that constraints from ore grade (i.e. the ratio of mineral in ore) are neglected, because ore grade should have been considered in mining plans and guaranteed by ore grade control (e.g. face assaying, stockpile blending) in each shift. The costs of consumables (such as explosive, drill air, and drill water) are not considered as constraints, because they eventually have to be consumed regardless of their prices and should have been taken into account in the cut-off grade when making mining plans. The expense of electricity, which is predominantly determined by the ventilation system, is not included in the constraints because there has already been the commercially available technique of ‘ventilation on demand’ providing ventilation optimization. The consumption of fuel, which is mostly determined by vehicle mileage, is actually already involved in the constraint of working time. The operating time of mobile mining machines is determined by the workload and operating rate. Therefore, by minimizing the entire working time, it will reduce not only the waiting time of mobile machines, but also the moving distance from one face to another, which indirectly reduces the fuel consumption.

The essence for improving underground mining performance is basically a question of finding the extreme value of the objective function under a number of constraints. In view of the practical needs of the mining industry, the objective function is normally the entire working time of a certain workload, which is to find a schedule with the minimum working time to complete the desired workload. This is a Flexible Flow Shop (FFS) problem, but not the typical one which has been studied before.

The FFS problem can be solved by branch and bound algorithms [9] and by mixed-integer-linear programming [10]. Such a method provides an exact solution which guarantees optimality. However, the exact solution can only be derived from small-scale instances (two-stage production). For large-scale FFS problems, these approaches take considerable time to obtain a solution. Heuristic or metaheuristic algorithms are needed for solving the large and complex FFS problems as they are Non-deterministic Polynomial-time (NP) hard for all conventional methods [11].

Early studies of heuristic applications focused on the simplified FFS case with only two stages. The worst and average performance of algorithms was assessed for finding minimum makespan, based on Johnson’s rule [12][13]. It was concluded that the longest processing time dispatching rule gives better results than the shortest processing time rule for two-stage makespan problem [14]. A scheduling method was investigated to minimize the makespan in a static flow shop with multiple processors. The method first generates an initial permutation schedule and then uses the first-in first-out (FIFO) rule to schedule the processors [15]. Gourgand et al. [16] use simulated annealing algorithms to a realistic industrial FFS problem. Jin et al. [17] propose two approaches to generate the initial job sequence with identical parallel machines and use a simulated annealing algorithm to improve the job sequence. Nowicki and Smutnicki [18] consider a Tabu search algorithm to solve the FFS makespan problem. Genetic algorithms have been broadly used in many previous studies. A genetic algorithm was developed to generate job sequences with minimum makespan [19]. Another genetic algorithm was developed to minimize the makespan including sequence-dependent job setup times. It is used in all production stages, and outperformed for more than two-stage production [11]. Cheng et al. [20] address the rapidness/tardiness scheduling problem with identical parallel machines, by using a genetic algorithm. Ruiz et al. [21] also use a genetic algorithm for the permutation of FFS scheduling problem with sequence-dependent setup times. Serifoglu and Ulusoy [22] developed a heuristic algorithm to schedule several machines to simultaneously work for an operation of a stage to minimize the makespan.

These approaches are mainly concerned with processing industries (e.g. textile, automobile assembly, printed circuit board manufacture, painting and packaging) which have multi-stage production with parallel machines at each stage. The purposes of the approaches are to increase the overall capacity or to balance the capacities of some stages, or to eliminate/reduce the impact of bottleneck stages. However, these approaches do not consider the distance between working places. Furthermore, they do not assign machines into different geographic areas; therefore, machines can work near to each other. However, this can risk the safety at an underground mining operation. For example, it is not recommended to have two drill rigs working close together. It is not possible to develop one single algorithm to obtain the schedule. It is required to develop and combine several algorithms based on real-world mining practice to cover the entire underground mining process.

### 3.1 Sequencing Algorithm

In underground mining, the difficulty of scheduling the different underground mining operations is to optimally assign many various tasks for many mobile mining machines. Unlike the conventional method which is to decide whether the next operation should work immediately or wait, a sequencing algorithm is developed with a compact and agile code structure. There has been a method using a search of a critical path in a directed graph [23]. However, this method does not consider the distances and moving time between working faces when determining the critical path, which can lead to an incorrect result.

The operations of the mobile mining machines at each working face are in series, while many working faces are processed by machines in parallel. Constrained by an underground environment, the underground mining operations mainly focus on improving the efficiency of the serial operations of several sequential machines, instead of improving the efficiency of the loader-truck cycle as in surface mining. The sequencing algorithm is applied to find the minimum timespan for serial operations under specific workloads. The timespan is from the start time of the first machine at the first face to the end time of the last machine at the last face.

For given faces f_1_ to f_n_ (1…n is face ID, n faces in total), and machines m_1_ to m_k_ (1…k is machine ID, k machines in total), assuming each face to be processed in the sequence from m_1_ to m_k_. The permutation of the sequences of the faces is n!. Therefore, there will be n! timespans (T). Minimum timespan is min(T_1_, T_2_, … T_n!_), which determines the optimal sequence of the working faces.

Three matrices are constructed:

$$\begin{array}{l} \text{Matrix of end time}\;\;\;\;\;\text{Matrix of operating time}\;\;\;\;\;\;\;\text{Matrix of moving time}\\ \left[\begin{array}{ccc} {\text{et}}_{11} & {\text{et}}_{12} & \ldots \\ {\text{et}}_{21} & {\text{et}}_{22} & \ldots \\ \ldots & \ldots & {\text{et}}_{\text{n,k}} \end{array}\right]\;\;\;\;\;\;\;\;\;\;\;\;\;\;\;\;\;\;\left[\begin{array}{ccc} {\text{ot}}_{11} & {\text{ot}}_{12} & \ldots \\ {\text{ot}}_{21} & \ldots & \ldots \\ \ldots & \ldots & {\text{ot}}_{\text{n,k}} \end{array}\right]\;\;\;\;\;\;\;\;\;\;\;\;\;\;\;\;\;\;\;\;\left[\begin{array}{ccc} {\text{mt}}_{11} & {\text{mt}}_{12} & \ldots \\ {\text{mt}}_{21} & \ldots & \ldots \\ \ldots & \ldots & {\text{mt}}_{\text{n,k}} \end{array}\right] \end{array}$$

Each element in the matrix of end time is a time stamp, while each element in the matrices of operating and moving time is a time interval. The element e_ij_ represent when machine j stops working at face i, while ot_ij_ and mt_ij_ respectively represent how much time machine j spends to operate at or move to face i.

$$\text{T}={\text{et}}_{\text{n,k}}-\text{st}=\text{max}\left({\text{et}}_{\text{n,k-1}},\;{\text{et}}_{\text{n-1,k}}+{\text{mt}}_{\text{n,k}}\right)+{\text{ot}}_{\text{n,k}}-\text{st}$$ (1)

Where et_n,k-1_ —the end time of machine k-1 at face n

et_n-1,k_—the end time of machine k at face n-1

mt_n,k_—the moving time of machine k from face n-1 to face n

ot_n,k_—the operating time of machine k at face n

st—the start timestamp of the entire operations

At time 0, i.e. st = 0,

$$\text{T}={\mathrm{et}}_{n,k}=\;\text{max}\left({\text{et}}_{\text{n,k-1}},\;{\text{et}}_{\text{n-1,k}}+{\text{mt}}_{\text{n,k}}\right)+{\text{ot}}_{\text{n,k}}$$ (2)

Nesting an array **t** in each element of the matrix of end time, as **e**_**i,j**_ **[t]**. The array of **e**_**1,1**_ **[t]** has only one element with the value ot_1,1_+mt_1,1_. The pseudo code for T is:

if (!(i = n & j = k))

{

for (j<k) {e_i,j+1_ = e_i,j_+ot_i,j+1_; add e_i,j+1_ in **e**_**i,j+1**_ **[t]**;}

for (i<n) {e_i+1,j_ = e_i,j_+ot_i+1,j_+mt_i+1,j_; add e_i+1,j_ in **e**_**i+1,j**_ **[t]**;}

}

if (i = n & j = k) T = **e**_**n,k**_ **[t]**.getMax

**Fig 5:**
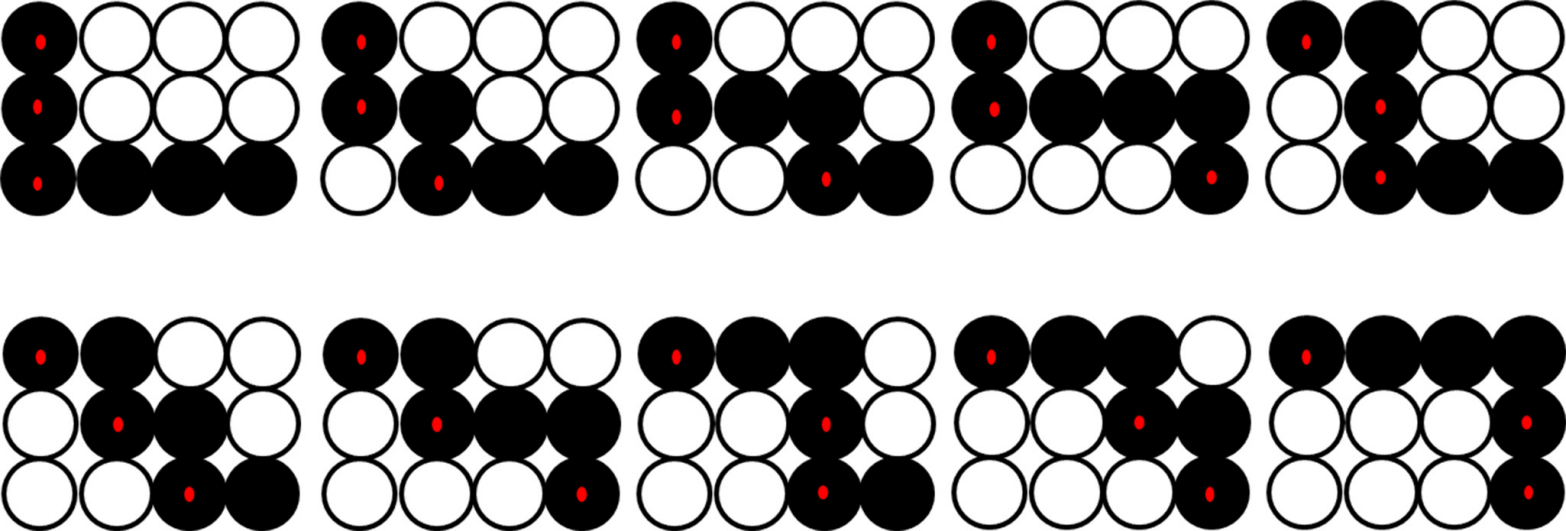
Example diagram of obtaining T (hollow dots—not counted, solid dots—counted, the solid dots with red core—also added on the relevant moving time).

For example, assuming a 3×4 matrix (which means 3 faces to be serially processed by 4 mobile mining machines) of operating time, Fig 5 demonstrates how to obtain T. The dots represent the elements of the matrix. The hollow dots represent the elements not counted, while the solid dots represent the elements counted and the solid dots with red core represent the elements which should also be added to the relevant moving time. There are ten summations from this matrix, and T is the maximum value of the ten results.

**Fig 6:**
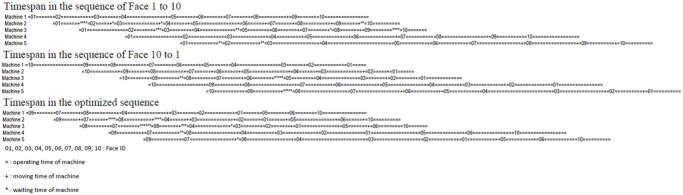
Comparison of timespans with different sequences.

Suppose that there are 10 faces (Face 1 till 10) to be processed by five mining machines (in the sequence of Machine 1 to 5). Fig 6 shows the outputs which do not use this algorithm, and the results using this algorithm. Comparing with the other two sequences (from face 1 to 10 and from face 10 to 1), the optimized sequence (in the sequence of face 9-7-8-4-3-2-1-5-6-10) has the least timespan to complete the assigned operations.

### 3.2 Grouping Algorithm

**Fig 7:**
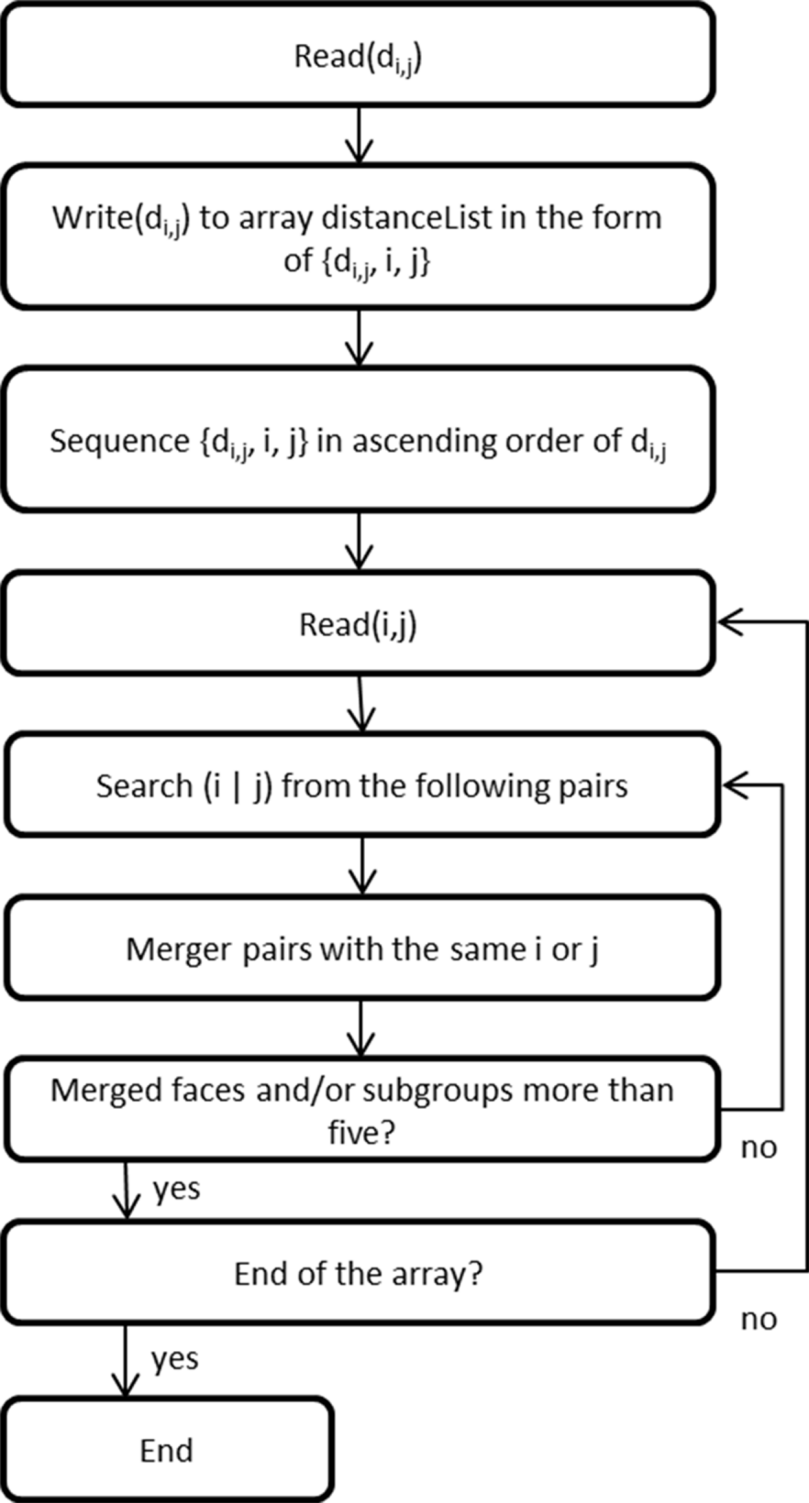
Flow chart of grouping algorithm.

In underground mines, there are normally many working faces required to be processed within a short period, e.g. one week. This can lead to a great number of permutations of sequences of the working faces. The computing workload can be too heavy for computers to rapidly obtain the result, or they may fail to solve the problem due to stack overflow in RAM. In the case of 10 working faces, there are 10! (= 3,628,800) permutations. One solution for this is to increase hardware capacity and/or have cache on hard disk. Another solution is to process fewer permutations. In the case of 10 working faces, if they can be divided into two groups, there are 5!+5! (= 240) or 6!+4! (= 744) permutations, which significantly reduces the computing time and workload. Therefore, grouping algorithm is used to divide the working faces into a number of groups. It can help to reduce the computing workload of the sequencing algorithm and the computing time. The basic principle of grouping is to group the faces which are relatively close, based on their distance d_ij_ (d_i,j_ is the path length between two working faces i and j). In view of the computing capacity of permutation, the maximum number of group, faces and sub-groups in one group, and faces in one sub-group is empirically set as five which can certainly be changed according to other users’ experience and hardware. After the first-round grouping, if there are more than five groups and faces left, the faces and groups will be grouped further, with a maximum of five faces or sub-groups in one group. This process will continue until there are not more than five groups. The algorithm is briefly described in Fig 7.

**Fig 8:**
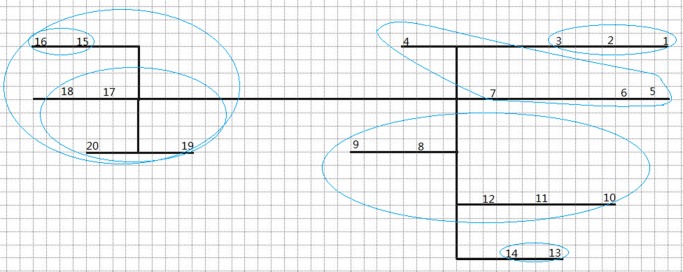
Grouping result of a conceptual vertical long-side section of an underground mine (number: Face ID; black line: path; blue circle: group and sub-group).

Fig 8 gives the result of this case by using the above grouping algorithm. It illustrates a conceptual vertical long-side section of an underground mine. The black lines represent underground paths; the numbers represent different working faces; the blue circles represent different groups. In each group, there are less than five faces or sub-groups. The faces and sub-groups are grouped according to the distances from each other. Combining the grouping algorithm and the sequencing algorithm can significantly reduce the computing work load. In Fig 8, the faces are first grouped into six groups, and there are two groups (15, 16; and 17, 18, 19, 20) being grouped in one group. Then the sequencing algorithm will find the optimized sequence of faces in each group and sub-group. Next the sequencing algorithm will find the optimized sequence of sub-groups within one group, and, finally, find the optimized sequence of groups in order to obtain the minimum timespan.

### 3.3 Machine Set Algorithm

**Fig 9:**
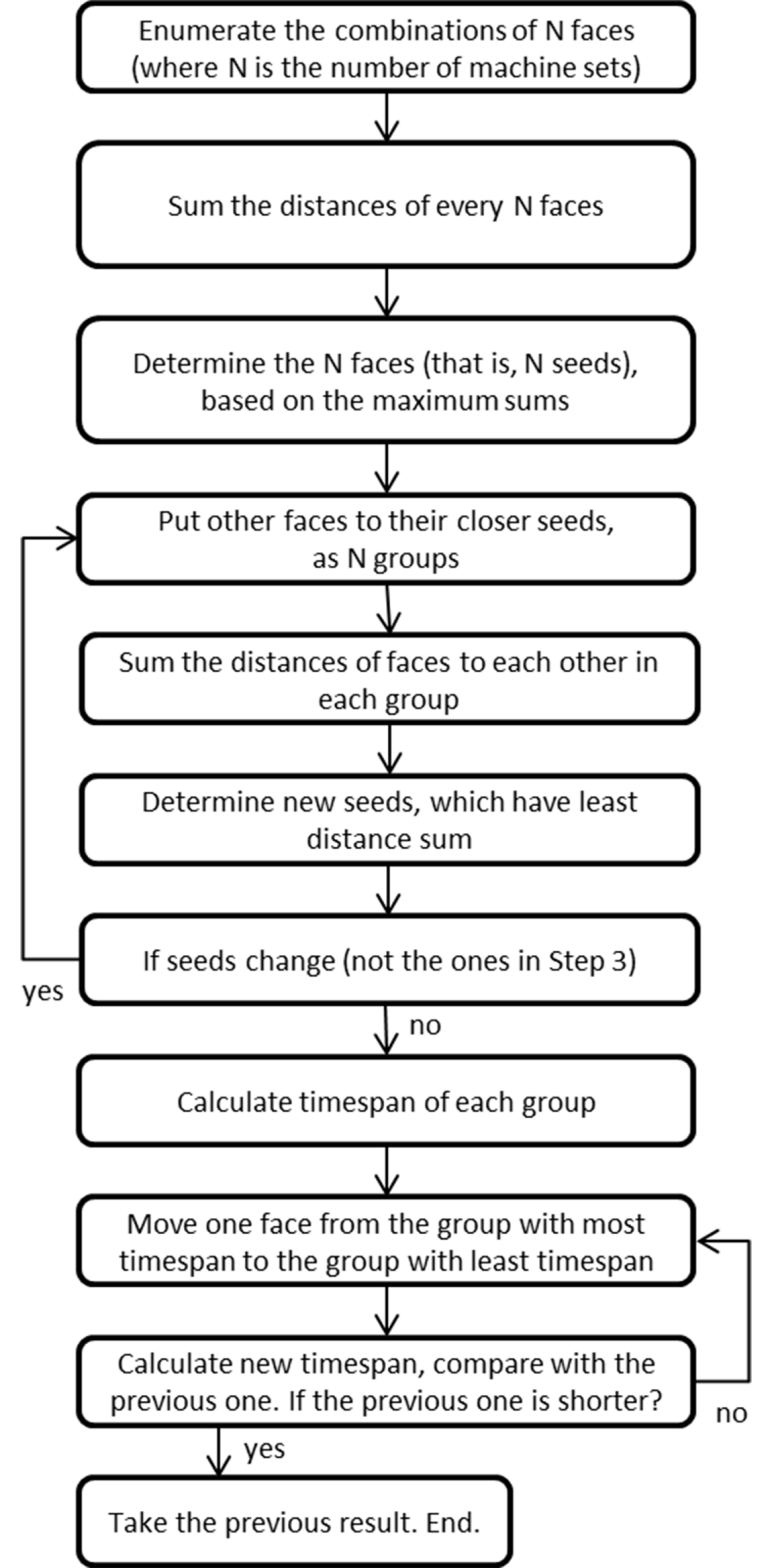
Flow chart of machine set algorithm.

**Fig 10:**
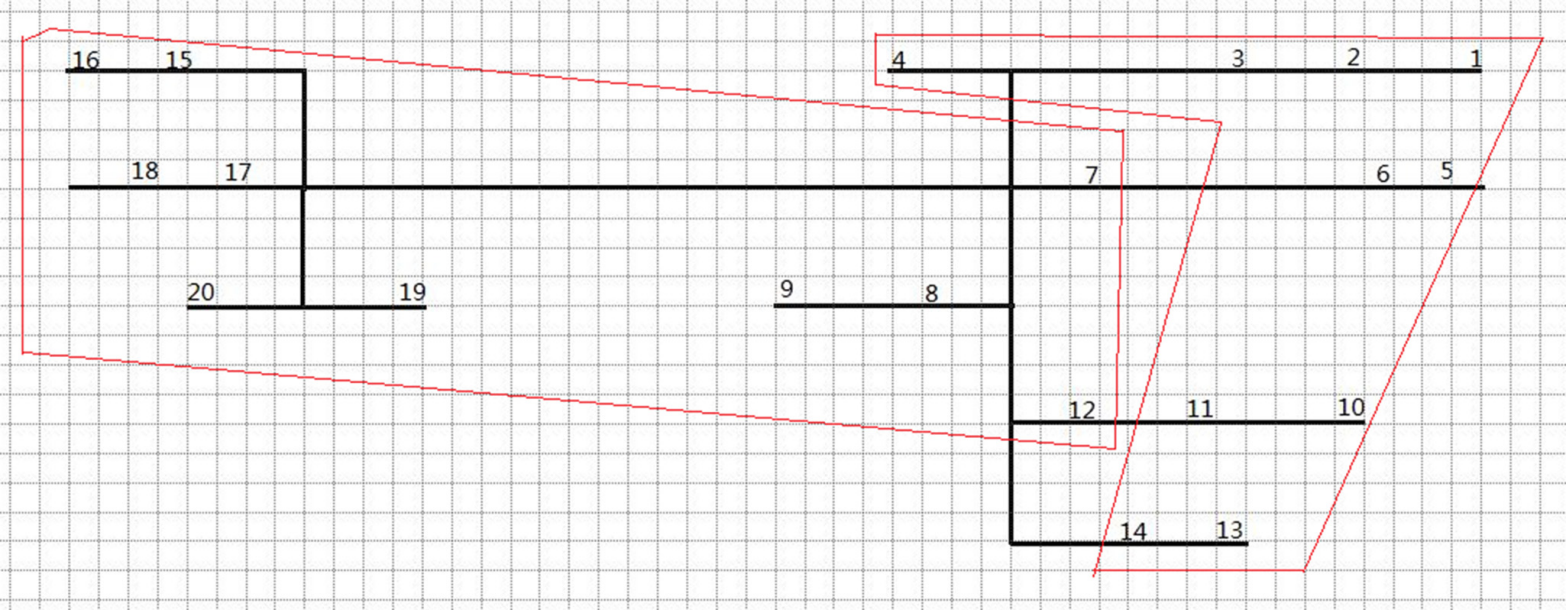
Clustering result of two machine sets (number: Face ID; black line: path; red border: mining area).

**Fig 11:**
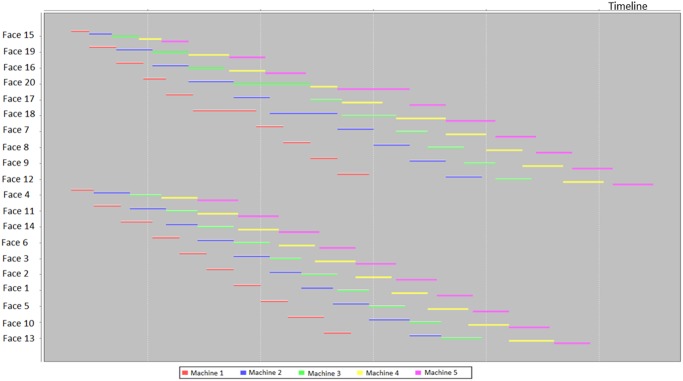
Scheduling result of two machine sets.

The machine set means one set of single mobile mining machines which operate in sequence, such as one scaler, one shotcreter, one bolter, one blast-hole driller, and one explosive charger. The serial operation of a single mobile machine is different from the cyclic operation of loading-hauling-dumping (loader-truck) which can have several machines at one face; therefore, the mucking cycle cannot be included in this algorithm. The components of machine set may vary in different underground mines. In large and medium mines, there are often several machine sets, which are used to improve the excavation/production rate and be backup for each other. The machine set algorithm is briefly given in Fig 9. This algorithm is used to assign different machine sets to different mining areas if there are more than one set. The objective of this algorithm is to assign the machine sets to obtain the minimum overall timespan. This algorithm first clusters faces into different mining areas, according to the distances between faces. The number of clusters is determined by the number of machine sets. Then, it calculates the timespan of each mining area respectively, and moves one face from the mining area with the longest timespan to the mining area with the shortest timespan, until the new overall timespan gets longer than the previous one. When calculating the timespan, this algorithm will first invoke on the function of the grouping algorithm to group the faces within different mining areas, and then invoke on the function of the sequencing algorithm to obtain the minimum timespan for that mining area. Figs 10 and 11 respectively demonstrate the clustering and scheduling results in the case of two machine sets, based on the assumed case in Fig 8.

### 3.4 Machine Sharing Algorithm

**Fig 12:**
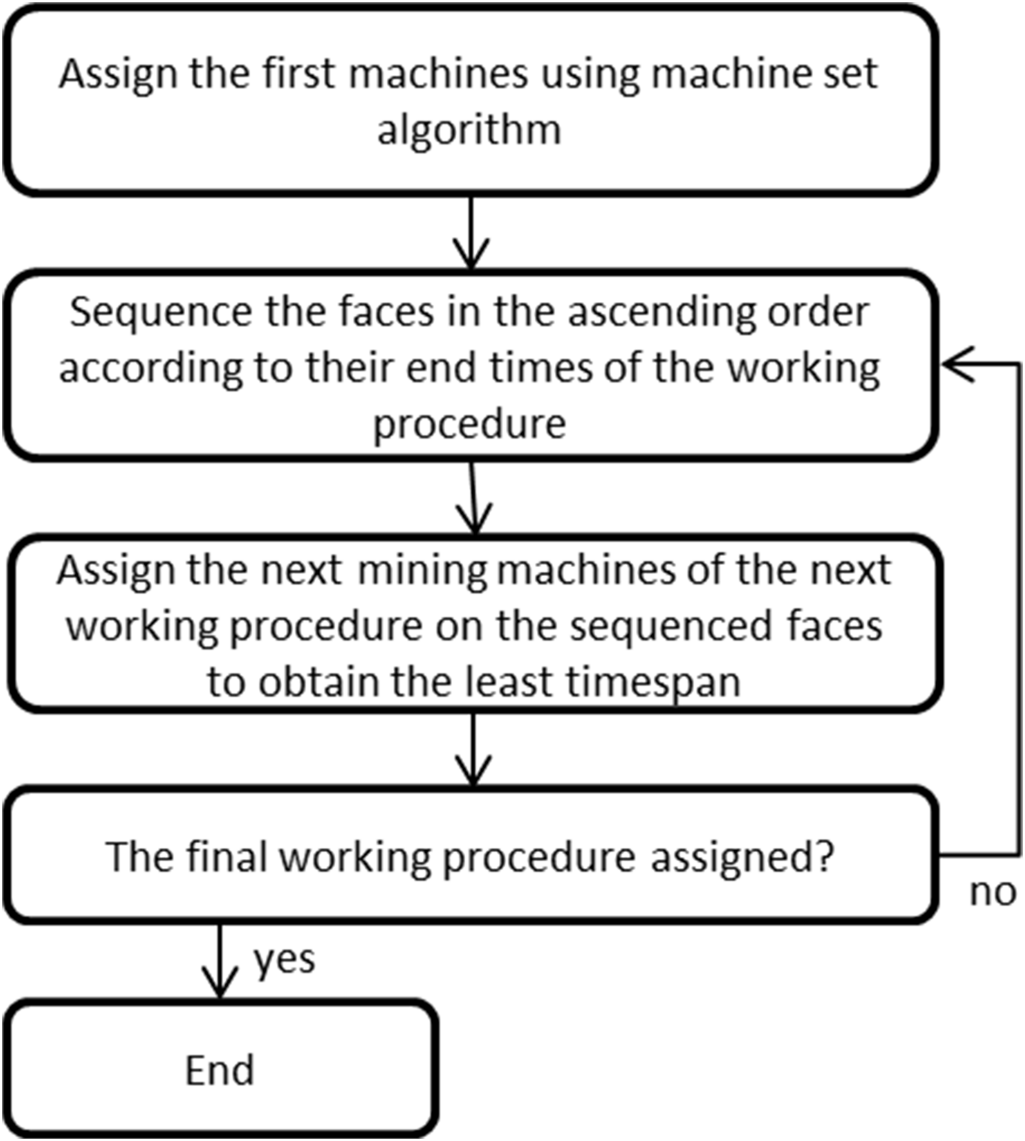
Flow chart of machine sharing algorithm.

**Fig 13:**
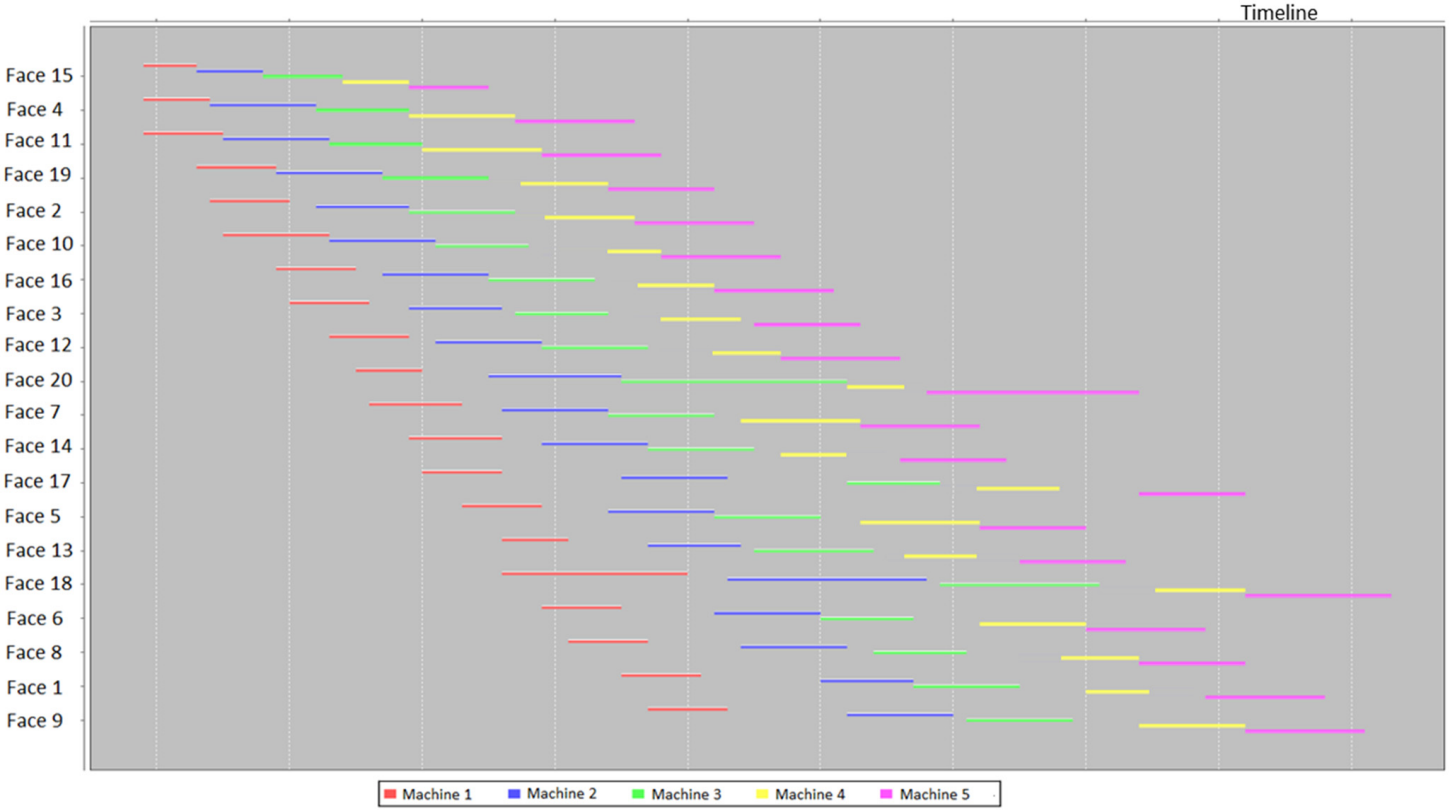
Scheduling result using machine sharing algorithm (assuming there are three sets of mining fleets, but one Machine 4 in one set is unavailable).

When there are more than one machine set, it is quite common to share mobile mining machines in underground mines in case some machines are not available to work for a certain period. This algorithm is designed to share mining machines, aiming to obtain the minimum overall timespan (Fig 12). It will invoke the machine set algorithm to assign the first mining machines which are in the first working procedure, then assign the second mining machines which are in the second working procedure, and so forth until the last mining machines which are in the final working procedure. Fig 13 demonstrates the scheduling result when using the machine sharing algorithm, in the case of three machine sets when one Machine 4 was unavailable.

## Application in Kittilä Mine

**Fig 14:**
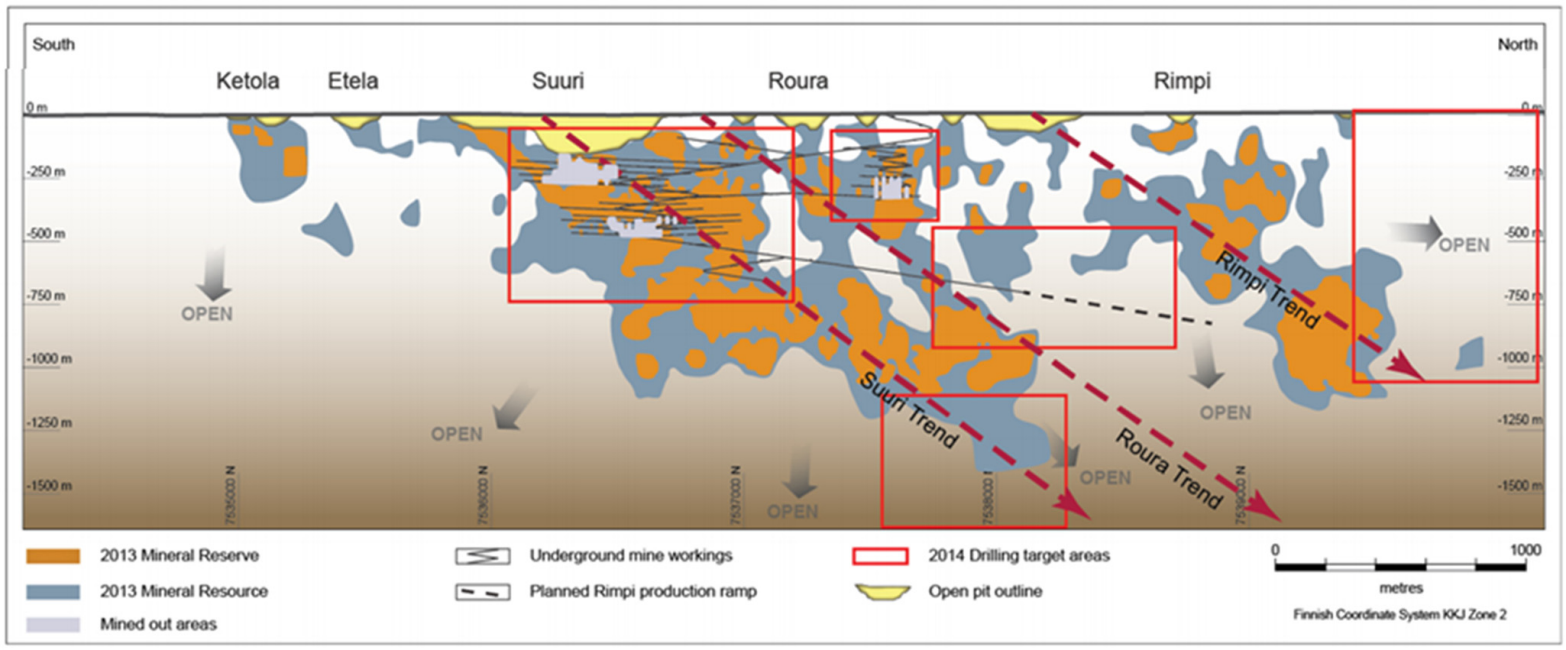
Cross section of Kittilä underground mine [24].

Kittilä mine is an underground gold mine located at Lapland, Finland. This mine is owned by Agnico Eagle Mine Limited. It started its mine production on 1 May 2009. The mine produces around 3,000 tonnes of ore per day, and it is targeted to produce 4,300 kilograms of gold in 2014. After this, the average production will be roughly around 4,700 kilograms of gold per year in 2015 to 2016. Originally Kittilä mine started as two open pits which are called Suuri and Roura. The underground mine started in October 2010. All of the open pits had already been mined out by November 2012. Now, only the underground mining exists. (Fig 14) The broken ore is hauled by truck to the surface and dumped at ore stockpile. The stockpile will feed the crusher which is next to the processing plant.

The Kittilä mine has two types of equipment fleet. They are the excavation fleet and the production fleet. The excavation fleet is used for constructing tunnels and openings in the underground. The production fleet is the fleet that is used to construct the stopes, in order to produce ore from the ore body.

The underground mining is run according to a weekly plan. There are weekly plans for excavation and production respectively. Normally the excavation includes more operations of machines than mine production does. In this case study, a weekly plan of excavation for the time period between 4 September 2013 and 10 September 2013 was used. The weekly plan mainly showed the headings which should be excavated in this week. The rest of the parts in the weekly plan showed information such as the total remaining material, total remaining excavation meters, and attention that should be given to certain tunnels (for example the risk of roof collapse and unavailable access of path).

In order for the mine to create the weekly plan, it first starts from the longest horizon of the mine plan, which is the Life-Of-Mine (LOM) plan. From the LOM plan, an 18-month plan is derived. After that, a monthly plan is created according to the 18-month plan, and finally the weekly plan is created based on the monthly plan. The weekly plan is implemented at the field by communicating between the foreman and his crew. These foremen usually use their own experience and personal judgement to implement the plan and to handle unexpected events during the implementation. This could often become biased and subjective. This is where the decision support instrument could assist them, by making an optimized detailed plan and proposing solutions to possible problems to achieve the weekly plan target.

**Fig 15:**
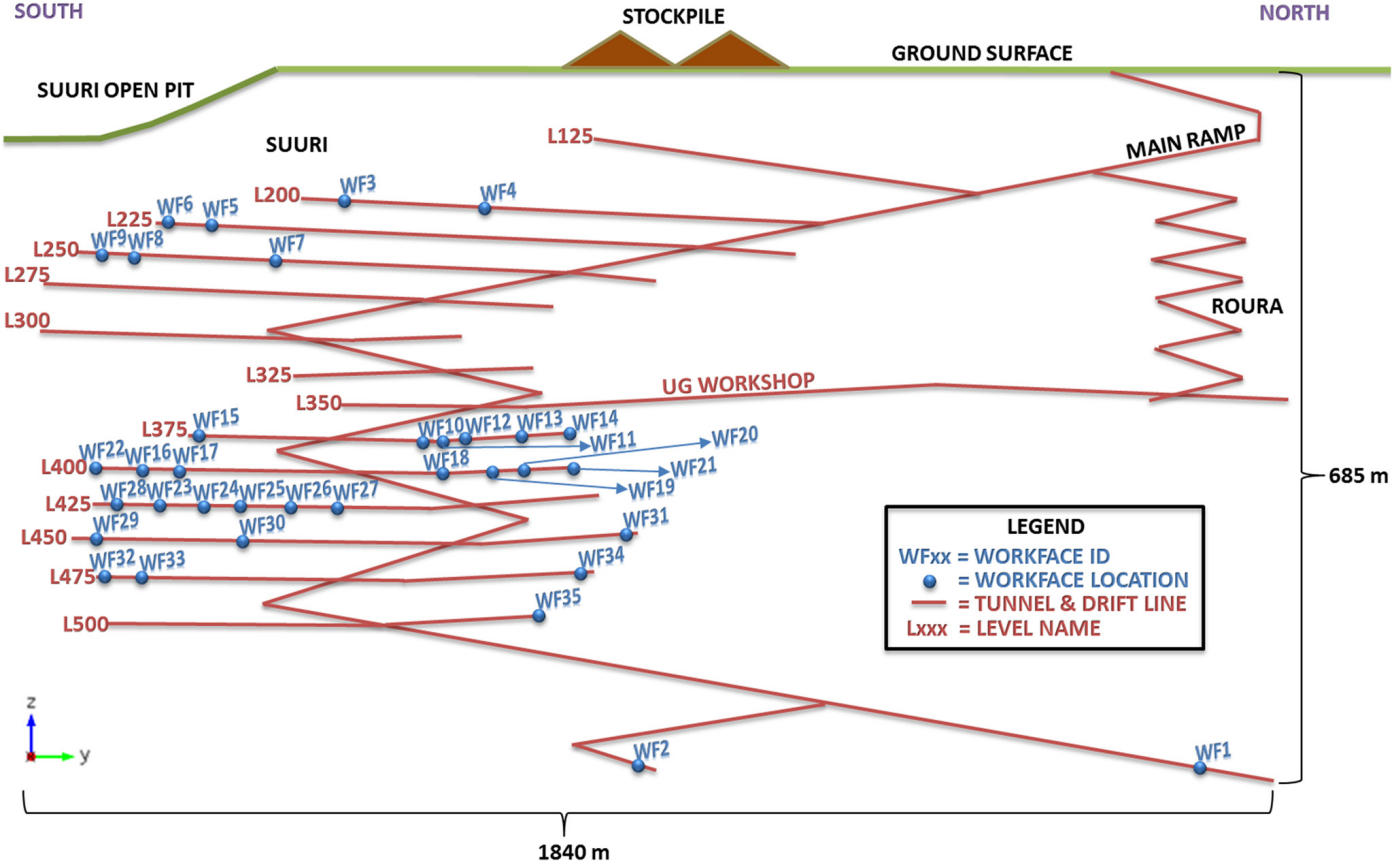
Layout of Kittilä mine with the locations of the working face used in the case.

The weekly plan of Kittilä during 4–10 September 2013 and the operating data of mobile mining equipment were used as the input data for the schedule optimizer. There were 35 working faces, 3 machine sets, and 7 types of machine (i.e. 7 working procedures at each working face). The workload at each working face was acquired from the Kittilä’s weekly plan, and the machine operating data were acquired from manufacturers’ manuals and experienced operators’ estimations. The locations of those 35 working faces are shown in the schematic layout of Kittilä mine in Fig 15. After the data were inputted into the schedule optimizer, the program was first run to obtain the scheduling based on the priority of each working face, and then followed by the lower prioritized working faces. The priority of each working face was set to the value of “1” in this case study.

**Fig 16:**
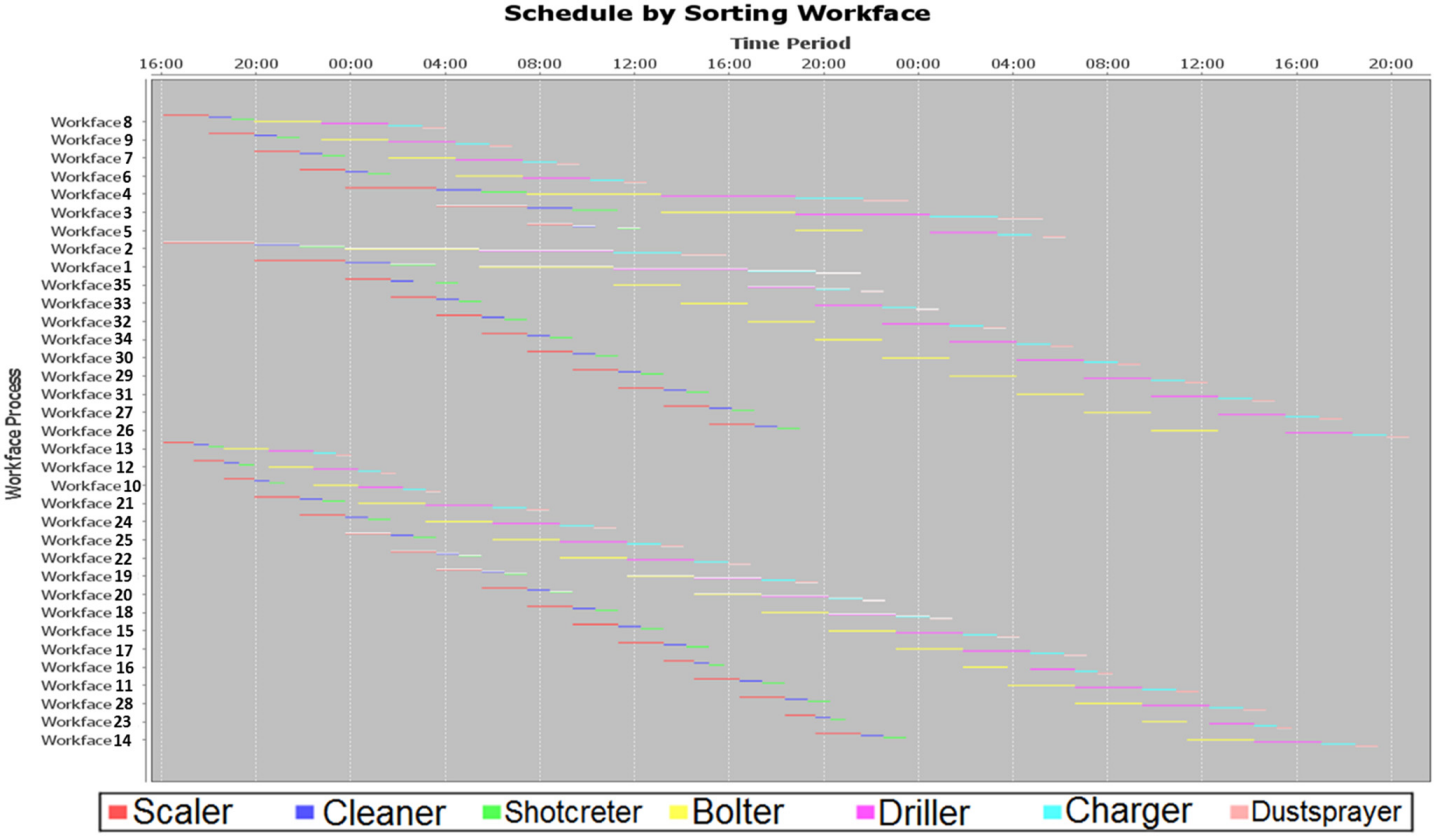
Scheduling output for the weekly plan of Kittilä mine.

After inputting and running the schedule optimizer by a CPU 1.7GHz and RAM 8GB laptop, a Gantt chart was produced within 20 seconds (Fig 16). The execution of the scheduling process uses the machine sets algorithm. The different machine sets respectively were assigned in different mining areas, i.e. Working Faces 3~9, Working Faces 1,2, 26~35, and Working Faces 10~25. And then the working faces in the mining areas are divided into smaller groups, by using the grouping algorithm. Next, the minimum timespan was found by invoking the sequencing algorithm. The Gantt chart shows that the entire mining process could be completed in 52 hours. It should be noted that the Gantt chart does not include shift-changing time, ventilation time, coffee and lunch time, which is around 18 hours for the two days. Furthermore, it does not include maintenance time of mobile machines because there were no broken-down machines reported in that week. Therefore, the optimized result gives a shorter period of circa 70 hours, which is less than one week when the mine did. The reasons of such discrepancy of the two working periods are the following: firstly, the schedule optimizer used the optimized sequence to schedule the jobs for each machine; secondly, the working parameters of machines were deterministic which can increase the error of this comparison; thirdly, there were idle times of crew and machines in the underground mining (therefore, there have been many applications of underground tracking and reporting developed). In view of the big difference between the optimized and actual result, it should be a considerable contribution of the schedule optimizing techniques.

Since there were no significant disturbances recorded in the mine during that week, five scenarios were created as a modification from the studied weekly plan of Kittilä in the previous stage to test the schedule optimizer. The various scenarios developed are shown in the Table 1, where each scenario was modified to be as realistic as possible. For example, Scenario 1 assumed that the access to Level 250 was suddenly cut off due to an unexpected roof collapse at this access. This event in Scenario 1 requires modifying the original data from Kittilä’s weekly plan. The modification was done by deleting several working faces 7, 8, and 9 from the original working face data set (i.e. the workload list and the distance matrix of working faces). The reason for deleting these is that these faces were located at Level 250. Therefore, there should not be workloads for mobile machines to work at these faces, but the mobile machines will still be able to work at other working faces which are accessible. Another example, Scenario 2 assumed that, in the second equipment fleet (or in other words, “machine set 2”), there was one charger broken down. This breakdown event made this equipment unavailable, therefore the equipment was simply “not available” in the input data (i.e. delete this machine from the machine list). Therefore, the three equipment fleets have to share two chargers. After the modification of the input data based on the different scenarios, the schedule optimizer was re-run for every scenario with its related input data. Each scenario could prolong or shorten the total working time. Table 1 also shows the working time given by the schedule optimizer. Based on the assumptions of the scenarios, the results are reasonable. In Scenario 1 and 4, the durations are shorter than in Scenario 0, because some workloads are removed. In Scenario 2 and 3, the durations are longer than in Scenario 0, because some machines are not available to work. In Scenario 5, it shows the same duration as in Scenario 0, because the initial locations are not sensitive in this case. Additionally, in the Kittilä mine, the foremen normally require around 10 minutes to make a decision for a new schedule. By using the decision support instrument, it only needs around 20 seconds to propose a new schedule, and the foremen just need to have a double check on the proposed schedule.

**Table 1:** Scenarios created for testing.

Scenario	Assumption	Duration
0	No modification	52h
1	Access to Level 250 was not available, thus eliminating workface #7-#9.	50h
2	One charger was not available in machine set 2.	56h
3	Only two machine sets were available.	66h
4	No scaling task at Workface 10, 15, 20 and 25.	51h
5	All machine initial locations were partially at workface 3 and 10.	52h

## Discussion

**Fig 17:**
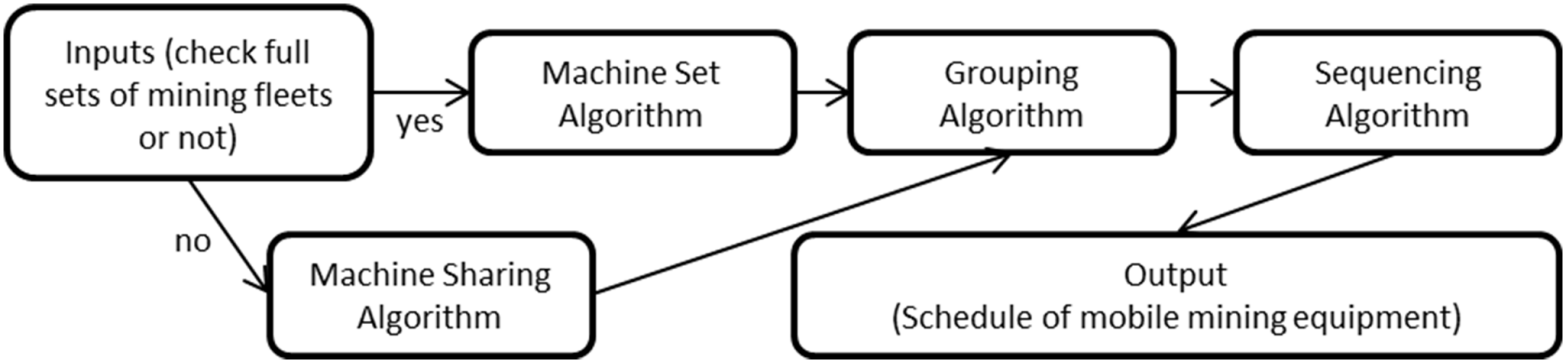
Relation graph of inputs, algorithms and outputs.

The four algorithms, i.e. sequencing, grouping, machine sharing, and machine set, should be coupled in practice. Fig 17 shows the relations of inputs, algorithms and outputs. First, all the related data should be input, and the machine set algorithm or machine sharing algorithm should be invoked, based on whether there are full sets of mining machines. Next the grouping algorithm is used to divide the faces into groups, and the sequencing algorithm is used to find the optimized sequence and generate the schedule of mobile mining machines.

It is noted that the algorithms designed for grouping, machine sharing and machine set are heuristic. In engineering, heuristics is an experience-based method to solve a problem where an exhaustive search is impractical or consumes too much time. Heuristics is intended to improve the efficiency of optimization within reasonable time, achieving a reasonable accuracy and precision. It neglects whether the solution can be proven to be optimum, but it usually produces a satisfactory solution within a proper time. Instead of obtaining all possible solutions, the algorithms of grouping, machine sharing and machine set are likely to produce reasonable outcomes in reasonable time. Combining the sequencing algorithm and the algorithms of grouping, machine sharing and machine set, it can first group the faces and then sequence the faces and sub-groups inside. It cannot guarantee that the result is the minimum timespan of the entire mining process, but it can guarantee that the timespans of faces and sub-groups inside of the groups are minimum and the result is reasonable and practical.

The mucking operation mentioned in this paper was not included, because it was a hot-spot of mining research, and there have been several software applications available in the market. They can assist miners in real time to manage the mucking operation and to obtain higher machine utilization and less queuing.

Additionally, because all the machines’ data are deterministic, there should be deviations from the schedule. In some cases where the deviation cannot be tolerated, the decision support instrument can reload the related data and rerun to obtain a new schedule. Besides, if there are priorities of faces which means some faces are required to be processed before others, the schedule optimizer will first handle the face with highest priority and then the next ones.

## Conclusion and Recommendations for Future

This research demonstrates the motivation for developing an intelligent application for optimizing the entire mining process in underground mining. It proposes a decision support instrument to assist miners to schedule mobile mining equipment, and elaborates the input/output and algorithms. The method is further illustrated by examples and a case study. Algorithms emphasize minimizing the timespan of the entire mining process for a specific workload within a certain period under given constraints. This instrument has potential to add practical value for the mining industry, especially for underground mining production and excavation. It gives clear indications to operators during what time which particular machine should work on which particular working face. It can be used to improve operational performance in more detailed machine scheduling, respond to unexpected events more promptly, and to forecast the budget of capital expense and operational expense more precisely.

The study in this paper is a preliminary step to future work. Firstly, this instrument will be installed and tested in Kittilä mine continuously with more cases, in order to verify and validate this approach. Time saving will be compared between the instrument’s proposed timespan and the actual timespan from the foremen’s schedule. Secondly, because there are high uncertainties in underground mining, the study will focus on the control of the underground mining process, to ensure the mining activities completed within their planned timeframe. Thirdly, because the approach is heuristic and near-optimal, it is necessary to obtain the real optimal schedules of several cases by using a better high-performing computer in order to thoroughly evaluate the approach with a real optimal solution.
